# Sacral Neuromodulation in the Management of Refractory Pediatric Lower Urinary Tract Dysfunction

**DOI:** 10.1002/nau.70365

**Published:** 2026-07-06

**Authors:** Megan A. Stout, Lauren E. Corona, Li‐Ching Huang, Tatsuki Koyama, Lindsay A. Gil, Shelly King, Benjamin M. Whittam, Seth A. Alpert, John C. Pope

**Affiliations:** ^1^ Department of Urology Vanderbilt University Medical Center Nashville Tennessee USA; ^2^ Department of Pediatric Urology Nationwide Children's Hospital Columbus Ohio USA; ^3^ Department of Biostatistics Vanderbilt University Nashville Tennessee USA; ^4^ Center for Surgical Outcomes Research, Nationwide Children's Hospital Abigail Wexner Research Institute Columbus Ohio USA; ^5^ Department of Pediatric Urology James Whitcomb Riley Children's Hospital Indianapolis Indiana USA

**Keywords:** bowel and bladder dysfunction, pediatrics, sacral neuromodulation, voiding dysfunction

## Abstract

**Introduction:**

Sacral neuromodulation is currently used in the pediatric patient population for refractory lower urinary tract dysfunction (LUTD). Evidence, however, is currently limited for institutional experiences of long‐term outcomes for sacral neuromodulation in the pediatric patient population.

**Objective:**

The objective of this study is to perform a retrospective review of a large multi‐institutional cohort of pediatric patients that underwent sacral neuromodulation (SNM) for refractory LUTD, focused on rates of symptom improvement, quality of life, and bowel‐bladder dysfunction measures following device implantation. Secondary objectives included assessing device complication rates and device removals due to failure or successful resolution of symptoms.

**Methods:**

We performed a retrospective cohort study of pediatric patients at three children's hospitals that underwent SNM device implantation for refractory LUTD. Patients 4–18 years of age who were diagnosed with refractory LUTD and managed at a participating institution with follow‐up were included. Outcomes were evaluated at baseline and post‐operatively after two‐stage device implantation at initial follow‐up and annually until device removal or final follow‐up.

**Results:**

From 2010 to 2022, a total of 205 children underwent SNM procedures at three pediatric hospital centers. 204 patients completed the two‐stage procedure for permanent device implantation with a median follow‐up of 3.4 years (1.3, 4.9). One hundred seventy‐eight patients reported partial (25%) or complete (54%) device response/symptom improvement by final follow‐up.

Comparison of patient‐reported validated questionnaires completed at baseline and by final follow‐up visit revealed significant improvement in median Pediatric Quality of Life Inventory (PedsQL) (*p* = 0.004) and Vancouver Nonneurogenic Lower Urinary Tract Dysfunction/Dysfunctional Elimination Syndrome bowel‐bladder dysfunction (BBD) scores (*p* < 0.001). Of those with any device complication (*n* = 43;21%), 19 patients required device revision, and 20 required complete device removal. Separately, twenty‐eight patients (13%) underwent device removal due to resolution of symptoms or treatment success at a median period of 5 years (4,7).

**Conclusion:**

SNM use demonstrated a majority of patients reporting at least partial subjective symptom improvement following device implantation across multiple institutions by final follow‐up. SNM is a viable treatment option for refractory LUTD in the pediatric patient if willing to undergo an invasive procedure and in which thorough counseling of risks and benefits has occurred.

## 1 Introduction

The lower urinary tract of the genitourinary system is regulated by a complex, coordinated multi‐level neural network coordination and controlling the activity of the bladder detrusor muscle and urinary sphincter [1, 2, 3]. Any dysfunction, imbalance, or lack of coordination of these neural signals can result in a myriad of lower urinary tract symptoms (LUTS), including urinary urgency, frequency, weak stream, intermittency, incontinence, retention, or dysuria as well as concurrent gastrointestinal symptoms [4, 5, 6, 7, 8]. The International Children's Continence Society (ICCS) describes and terms this clinical presentation under the overarching category of lower urinary tract dysfunction (LUTD) [9]. In 17%–22% of all children, non‐volitional or socially unacceptable voiding persists beyond age five, characterizing LUTD [10]. In the pediatric population, initial treatment for LUTD involves a combination of standard urotherapy (defined by ICCS as first‐line, non‐surgical, non‐pharmacological treatment for LUTD focusing on education, behavioral changes, lifestyle adjustments, symptom tracking via voiding diary, aimed at normalizing bladder function) and pharmacological agents with success rates ranging from 60% to 80% [11, 12]. However, a small cohort of patients have persistent symptoms despite interventions, and these refractory cases may benefit from additional therapy such sacral neuromodulation.

Sacral neuromodulation (SNM) has been United States Food and Drug Administration (FDA) approved for adults for over 20 years for the indications of non‐obstructive urinary retention, refractory overactive bladder, and fecal incontinence. Evidence, however, is lacking regarding symptom improvement and device outcomes of SNM in children [13]. Further investigation is required to characterize symptom progression and quality‐of‐life outcomes over the course of SNM device use, as well as rates of device complications, revisions, and removals (due to device failure or symptom resolution) to inform the management of refractory LUTD in children.

Our sacral neuromodulation consortium is a multi‐institutional network and database established to better understand and advance the practice of managing pediatric LUTD with SNM. The objective of this study is to perform a retrospective review of a large multi‐institutional cohort of pediatric patients that underwent SNM for refractory LUTD, and to assess changes in bowel and bladder symptoms and quality of life measures following device implantation. Secondary objectives included assessing device complications and removals due to device failure (mechanical and irresolution of symptoms) or successful resolution of LUTD symptoms.

## 2 Materials and Methods

### 2.1 Patients

Following institutional review board approval, we identified 205 patients < 18 years of age who underwent SNM device implantation for refractory LUTD at a consortium‐designated institution between 2010 and 2022. For the context of this study and documentation purposes across institutions, patient selection was designated purely symptom‐based (description of a constellation of symptoms was utilized and not individual diagnoses alone). Patients were deemed refractory upon clinical assessment after failure of alleviation of symptoms despite trial of conservative therapies including any combination of behavioral or medical therapy including adequate hydration, appropriate toilet posturing, timed or double voiding, treatment of constipation, pelvic floor physical therapy, biofeedback, and medication trial (anti‐muscarinics, beta‐3 agonists, or alpha blockers). Evaluation prior to device implantation included baseline video urodynamic evaluation (*n* = 192) and spinal MRI (*n* = 164) to rule out underlying neurologic abnormalities contributing to dysfunction, and was left up to the discretion of the individual provider when more objective preoperative data was necessary therefore, practices between institutions were not uniform. Imaging and urodynamic reporting was reviewed by each individual provider at participating institutions. Urodynamic interpretation was largely individualized, but for data collection purposes was categorized into whether there was evidence of pelvic floor overactivity, detrusor overactivity, smaller‐than‐expected‐capacity bladder, underactive bladder or detrusor underactivity, or otherwise normal study findings for each patient.

### 2.2 Procedure

Patients underwent placement of a sacral neuromodulation device (Interstim II, Medtronic, Minneapolis, MN). Device was placed by a pediatric urologist. In general, a two‐stage technique was used. Transforaminal access to the S3 nerve root was performed under fluoroscopy with a tined quadripolar lead implanted after confirmatory neurologic response. The lead was then connected to an external pulse generator and programmed over a trial period of 2 weeks. If patients experienced a > 50% improvement in symptoms defined by mix of subjective report of patient and family as well as interpretation of voiding diary data when available, then placement of the implantable pulse generator (IPG) was performed in the operating room. Minor variations in technique were permitted at the discretion of the treating surgeon based on clinical judgment or patient‐specific factors.

The primary outcome was subjective patient or caregiver‐reported improvement at last follow‐up, as documented in the electronic medical record (EMR) with scoring of validated questionnaire results completed at baseline and at final follow‐up, which included Pediatric Quality of Life Inventory (PedsQL) and Vancouver Nonneurogenic Lower Urinary Tract Dysfunction/Dysfunctional Elimination Syndrome bowel‐bladder dysfunction (BBD) scoring. Secondary outcomes included assessment of device complications or device removal (due to mechanical/symptomatic treatment failure or success) defined by physician documentation reporting in the EMR.

### 2.3 Data Collection

Demographics, indication for device implantation, and prior therapies were abstracted. Symptom improvement at follow‐up was determined by review of available subjective reporting utilized in clinic documentation notes to the provider when a likert‐like scale of terms was provided describing “a little” or “a lot” better or worse compared to baseline. For clinical applicability, these responses were then translated into standardized terminology according to the Standardization Committee of the International Children's Continence Society for lower urinary tract function terminology reporting, which utilized “Complete response” as 100% improvement, “Partial response” as 50%–99% improvement, and “No response” as < 50% improvement [9]. Therefore, those with response of “a lot” better were determined to be complete response, “a little better” were deemed to be partial response, and those with no change, a little worse, or a lot worse were categorized as no response.

Symptom improvement was further characterized via the two validated questionnaires (BBD to assess BBD severity and health‐related quality‐of‐life parameter assessment via the PedsQL) administered to patient or caregiver present [14, 15]. The BBD questionnaire consists of a 14‐item 5‐point Likert scale questionnaire with scores ranging from 0 to 52, with higher scores indicating more severe BBD [14]. The 23‐item PedsQL questionnaire uses a five‐point response format to assess physical, emotional, social, and school functioning in children, with items reverse‐scored and transformed to a 0–100 scale, where higher scores reflect better health‐related QOL [15].

Questionnaires were administered to every patient or caregiver at baseline prior to intervention and every follow‐up visit with urology thereafter – participation in questionnaire completion was optional. Follow‐up protocols were non‐standardized between institutions; however, follow‐up visits typically occurred within 1 month after device implantation and then annually until device removal or lost to follow‐up. Patients who experienced sustained symptom improvement for at least 6 months were offered elective explantation, which was recorded as removal due to treatment success. Prior to this elective explantation, an intentional trial period with the device turned completely “off” was utilized for several weeks (time variable depending on treating institution) to ensure that symptoms remained controlled. This was distinguished from device removal for complication or failure (mechanical or lack of symptom improvement). Postoperative urodynamics were performed at the discretion of the provider on a case‐by‐case basis (such as in failure of symptom improvement or new onset urinary retention).

All data obtained from participating institutions were entered into a secure REDCap database [16].

### 2.4 Statistical Analysis

Due to non‐standardized follow‐up protocols among institutions, this study focuses on data from the first and final follow‐up visits. Demographics, treatment history, and subjective reported symptom improvement data were summarized using medians (quantiles) and frequencies (percentages). A paired t‐test was used to compare baseline, first, and final follow‐up PedsQL and BBD scores. Statistical significance was assessed at two‐sided 5% level. All statistical analyses were performed using R statistical software version 4.4 [17].

## 3 Results

From 2010 to 2022, 205 children underwent SNM procedures at three pediatric hospital centers. 141 (69%) of these patients were female, 186 (91%) were White/Caucasian, and 125 patients (61%) were insured through private insurers (Table 1). The majority of patients had tried behavioral modifications (*n* = 144; 70%) and anti‐muscarinic medication (*n* = 175; 85%) prior to device implantation, while less than half of patients utilized pelvic floor physical therapy or biofeedback (*n* = 95; 46%). The most common subjective voiding complaints included incontinence (56%) and urinary urgency/frequency (24%). Preoperative urodynamics were performed in 192 (94%) of the patients, with detrusor overactivity being the most common abnormality noted (*n* = 109; 57%), while 11% of patients had a normal study. Baseline PedsQL score was obtained in 45 patients, with median value of 78 out of 100 (Median quartile 65, 90). Baseline BBD score was obtained in 106 patients, with median value of 21 out of 52 (17, 28) (Table 2). Median age at the time of 1st stage surgery was 10 years (8, 13), and median BMI was 18 (16, 23).

**Table 1:** Patient demographics (*N* = 205).

Characteristic	*N* (%)
Treating Institution	
A	84 (41)
B	69 (34)
C	52 (25)
Sex	
Female	141 (69)
Male	64 (31)
Race	
White or Caucasian	186 (91)
Black or African‐American	10 (5)
Unknown/Not reported	5 (2)
Asian	4 (2)
Insurance type	
Public (Medicaid, Medicare)	62 (30)
Private	125 (61)
Self‐pay	12 (6)
Other	6 (3)

**Table 2:** Treatment history and baseline characteristics.

Clinical characteristic	*N* (% or Median Quartile) [Number of patients]
Prior Treatment*	[205]
Behavioral modification	144 (70)
Anti‐muscarinic	175 (85)
Alpha1‐antagonist	90 (44)
Physical therapy/biofeedback	95 (46)
Predominate voiding complaint	[205]
Urinary retention	13 (6)
Urgency/frequency	50 (24)
Incontinence	115 (56)
Recurrent UTIs	7 (3)
Other	20 (10)
UDS obtained pre‐operatively	[205]
Yes	192 (94)
No	13 (6)
UDS finding	[192]
Normal	21 (11)
Pelvic floor overactivity	17 (9)
Detrusor overactivity	109 (57)
Small capacity bladder	37 (19)
Underactive bladder/detrusor underactivity	8 (4)
Age at 1st stage	[205]10 (8,13)
BMI at 1st stage	[161]18 (16,23)
Days from 1st to 2nd stage	[204]14 (12,15)
Baseline PedsQL Score	[45]78 (65,90)
Baseline BBD Score	[106]21 (17,28)

*Note:* * Responses were not mutually exclusive.

Abbreviations: BBD, Vancouver Nonneurogenic Lower Urinary Tract Dysfunction/Dysfunctional Elimination Syndrome bowel‐bladder dysfunction; BMI, Basal Metabolic Index; PedsQL, Pediatric Quality of Life Inventory; UDS, Urodynamic Study.

A total of 204 of the 205 patients underwent a second‐stage procedure for IPG of SNM placement. One patient did not have at least 50% improvement in urinary symptoms following trial period of first stage procedure, therefore the patient did undergo IPG placement. Median follow‐up was 3.4 years from the time of first‐stage implantation (1.3, 4.9). Seventy‐nine out of 204 individuals (39%) had at least 4 years of follow‐up, while 3 (1%) of patients had at least 8 years of follow‐up. Of the cohort, 178 patients (79%) self‐reported feeling “a little” (25%) or “a lot better” (54%) after device implantation by last follow‐up. According to ICCS reporting criteria, this translates to 25% of patient cohort with partial response and 54% with complete response following device implantation.

In those with both baseline and postoperative completed PedsQL (*n* = 19) and BBD (*n* = 67) questionnaires, there was significant improvement in PedsQL scores (median improvement by 10 points, median quartile 0,26; *p* = 0.004) and BBD scores (median improvement by −9 points, median quartiles −14.5, −2; *p*< 0.001) by last follow‐up (Table 3).

**Table 3:** Postoperative follow‐up data N (% or Median Quartile) [Number of patients].

	First follow‐Up	Final follow‐Up
Time from 2nd stage procedure to follow‐up (years)	0.1 (0.0, 0.3) [204]	3.5 (1.3, 4.9) [204]
Age (years)	10 (8, 13) [205]	14 (11, 16) [205]
Patient perceived improvement	[199]	[195]
A lot better	100 (50)	106 (54)
A little better	78 (39)	48 (25)
Remains the same	17(9)	24(12)
A little worse	4 (2)	13 (7)
A lot worse	0(0)	4(2)
Patient perceived improvement via ICCS reporting criteria [9]	[199]	[199]
Complete response	100 (50)	106 (54)
Partial response	78 (39)	48 (25)
No response	21 (11)	41 (21)
PedsQL change in score from baseline	3 (−4, 16) [34] *p *= 0.18	10 (0, 26) [19] ***p* = 0.004**
BBD change in score from baseline	−5.5 (−11.0, −1.0) [89] ***p* < 0.001**	−9.0 (−14.5, −2.0) [67] ***p* < 0.001**

Bold values indicate statically significant.

Abbreviations: BBD, Vancouver Nonneurogenic Lower Urinary Tract Dysfunction/Dysfunctional Elimination Syndrome bowel‐bladder dysfunction; PedsQL, Pediatric Quality of Life Inventory.

Forty‐three patients (21%) had documentation of any form of complication following device implantation, with 39 patients (19%) requiring surgical re‐intervention, including device revision (9%) or removal (10%). Device revisions were only completed for implantable generator or battery malfunction, or in the case of lead migration. The most common reason for complete device removal was device/wound infection (*n* = 6) or no improvement of symptoms (*n* = 5). Separately, twenty‐eight of the patients (14%) underwent device removal due to resolution of LUTD symptoms (success) at a median time of 5 years (4,7 y) from time of implantation (Table 4).

**Table 4:** Complications, revisions, and removals of device by final follow‐up (*N* = 205).

	*N* (% or Median Quartile)
Any complication following device implantation	43 (21)
Complication requiring surgical intervention	39 (19)
Surgical intervention required for:	
Revision	19 (9)
Removal for failure	20 (10)
Reason for surgical removal for failure:	
No improvement of symptoms	5 (2)
Side effects of device	2 (1)
Patient non‐compliance	1 (0.5)
Wound/device infection	6 (3)
Other	6 (3)
Device removal due to successful treatment	28 (14)
Time from 1st stage device implantation to successful removal (years)	5 (4,7)

## 4 Discussion

Despite the lack of FDA indication for SNM use in children, sacral neuromodulation remains a recognized therapy for refractory LUTD in pediatric patients. We aimed to contribute to the current body of evidence in this patient population with our multi‐institutional review of the experience of 205 patients. We found that 79% of patients demonstrated partial (25%) or complete (54%) device response by final follow‐up. When assessing objective parameters of device experience, patients that successfully completed validated questionnaires demonstrated a significant improvement in overall quality of life and BBD‐related questions by last follow‐up. Of note, 21% of patients experienced some form of complication following device implantation in a median follow‐up period of 3.5 years, with 9% of patients requiring device revision sometime in their treatment course and 10% requiring complete device removal. Twenty‐eight patients had device removal due to treatment success at a median time of 5 years.

Previous studies have aimed to qualify and quantify the degree of symptom improvement to describe SNM device efficacy in pediatric patients. Schober et al evaluated 26 patients in 2015 who underwent SNM, with their symptoms measured pre‐ and post‐operatively by voiding questionnaires and repeat urodynamic studies. Voiding dysfunction scores improved significantly for 23 of the patients on post‐operative questionnaires. Additionally, post‐operative urodynamic studies demonstrated a significant decrease in mean number of uninhibited detrusor contractions and a significant reduction in maximum detrusor pressures during filling phases [18]. Assessment of patients undergoing sacral neuromodulation using validated quality of life and bladder dysfunction questionnaires has also previously been studied [19]. Stephany et al prospectively enrolled 14 patients in their evaluation following generator placement. Patients had significant improvement in NLUTD/DES scores, psychosocial, and overall total quality of life scoring. Our utilization of the BBD and PedsQL validated questionnaires aimed to assess the interplay of a device implant and its translatable effects on other bodily health systems and overall quality of life [14, 15]. We also aimed to qualify and quantify these symptom data available from our patient cohort via subject patient/parent reporting and validated questionnaire administration at baseline and subsequent follow‐up. In our experience, one hundred seventy‐eight (89%) patients reported at least some improvement in their symptoms since device implantation by first follow‐up. We also saw statistically significant improvements in both validated questionnaires utilized in patients from baseline to final follow‐up assessment for those that completed these successfully.

Device placement is not without risk of complication. Concerns with utilization of implantable device implantation in a pediatric patient include lead migration due to patient linear growth, need for repeated magnetic resonance imaging (MRI), need for multiple surgical procedures for device revision or battery replacement, or prolonged fluoroscopy exposure [20]. One of the largest single‐institution studies conducted by Boswell et al. reported that of 180 pediatric patients that underwent permanent device placement, 154 total secondary surgeries were performed. There were 83 device revisions, with 89% of revisions performed for a non‐functioning device [21]. Permanent device removal was performed in 71 (39%) of patients, with 38% for unfavorable reasons (infection, pain, or no longer effective). We reported an overall complication incidence of 21% (*n* = 43) through a median follow‐up period of 3.5 years. Thirty‐nine (19%) of these complications required surgical intervention for device revision or removal. It is therefore critical for patient and parent understanding of these risks prior to surgical intervention. Informed consent should include discussion of the high rates of revision or reoperation and the potential for device infection or long‐term complications.

Surprisingly, 5 patients required device removal for no improvement in symptoms despite an initial successful trial period prior to second‐stage implantation. There are any number of potential reasons for this discrepancy in reporting: a change in subjective response in follow‐up, difficulty with device handling education or need for re‐programming if needed, or patient or parent non‐compliance for device function/evaluation. Without individually interviewing these 5 patients or if clinic documentation did not clearly denote reasoning why they reported no improvement in symptoms, we are limited by the retrospective design of this study. Understanding this discrepancy would be a great area of research and further analyzation.

The concept of potential SNM device removal due to treatment success is positive for patient, parent, and provider alike. The mechanism of SNM remains poorly understood; however, it is thought that stimulation at the sacral spinal cord may lead to neural remodeling of the spinal cord and brain [22]. This remodeling has been demonstrated only in adult populations with a review of functional MRI showing changes in brain activity once an SNM is activated [23]. Rensing et al described a pediatric cohort that had SNM explant due to “success” or “cure”, noting a significantly improved quality of life reported on questionnaires following device removal compared to those patients undergoing device explantation due to complications [24]. Boswell et al. also described their experience of pediatric patients that underwent device explantation due to symptom resolution or improvement. They noted that 44/154 (28%) patients had symptom improvement or resolution that was maintained during an intentional trial period with the device off, allowing device removal [21]. They also noted 74% of their patients by last follow‐up were either utilizing the device with good results, trialing the device “off”, or the device had been removed due to complete improvement. In our own cohort, twenty‐eight of the patients (14%) underwent device removal due to resolution of LUTD symptoms (success) at a median time of 5 years (4,7 y) from time of implantation. Both Boswell's work and our multi‐institutional experience together could be evidence to support that a small number of patients successfully undergo device explantation due to treatment success. This remains an area of further evaluation to elucidate the true mechanism of SNM neurologic central nervous system remodeling versus the natural course evolution of pediatric LUTD.

The study's limitations include those inherent to a retrospective design. Although standardization techniques were emphasized across participating institutions, variability exists in clinical pathway and surgical techniques performed, which could influence results and interpretations. We also did not routinely perform urodynamic studies in the post‐operative period across institutions, and choice of performance was largely dependent on provider discretion. Therefore, generalizability may be limited. We attempted to capture treatment success with subjective and objective parameters via patient reporting and validated questionnaire use; however, data collection was greatly limited by the lack of compliance in questionnaire completion at follow‐up. We also attempted to mediate the interpretability of subjective response reporting by translating these responses into ICCS reporting criteria terms, however, we understand that these terms are not directly translatable. This highlights the limitations of subjective reporting. We recognize the overall limitation of subjective reporting in general in pediatric clinical practice, especially in children who are less than school age, in which we are dependent upon parent proxy‐interpretation of patient symptoms. Given the observed subjective symptom improvements and the statistically significant improvements demonstrated in the limited number of patients who completed the questionnaires in our study, we hypothesize that broader utilization of these tools may yield similarly significant results. Despite these limitations, our data adds to the small body of literature describing the experience of SNM in the refractory LUTD pediatric patient population. We highlight our experience of one of the largest cohorts of pediatric patients that have undergone SNM device implantation in a multi‐institutional setting.

## 5 Conclusion

This large, multi‐institutional patient cohort provides a comprehensive clinical picture of the SNM experience and outcomes in children, highlighting evidence of partial or complete device response in 79% by final follow‐up. Ongoing evaluations at our participating institutions will allow for continued longitudinal assessment of SNM outcomes in order to adequately assess device durability and longevity. While our results revealed a high complication rate of 21% and an overall reoperation rate of 33%, it is noteworthy that nearly half of these reoperations were performed in the context of treatment success. Sacral neuromodulation remains a viable treatment option for refractory LUTD in the pediatric patient if willing to undergo an invasive procedure and in which thorough counseling of risks and benefits has occurred.

## Author Contributions

**Megan Stout:** data synthesis, manuscript writing. **Lauren Corona:** manuscript writing and revision. **Li‐Ching Huang:** data analysis. **Tatsuki Koyama:** data analysis. **Lindsay Gil:** data synthesis, manuscript writing. **Shelly King:** data collection, study design. **Benjamin Whittam:** data collection, study design. **Seth Alpert:** data collection, study design. **John Pope:** data collection, study design.

## Funding

The authors have nothing to report.

## Ethics Statement

There is no violation of ethics or integrity to be disclosed through this study. This study is IRB approved by all participating institutions. This study is IRB approved by all participating institutions

## Consent

Patient and parent consent was obtained at time of initial data collection for administration of validated survey data at each participating institution. Permission to reproduce material from other sources is available from the corresponding author upon reasonable request.

## Conflicts of Interest

The authors declare no conflicts of interest.

## Data Availability

The data that supports the findings of this study are available from the corresponding author upon reasonable request.
